# Mechanical ventilation: past lessons and the near future

**DOI:** 10.1186/cc11499

**Published:** 2013-03-12

**Authors:** John J Marini

**Affiliations:** 1 University of Minnesota, Regions Hospital MS11203B, 640 Jackson Street, St Paul, MN 55101, USA

## Abstract

The ability to compensate for life-threatening failure of respiratory function is perhaps the signature technology of intensive care medicine. Unchanging needs for providing effective life-support with minimized risk and optimized comfort have been, are now, and will be the principal objectives of providing mechanical ventilation. Important lessons acquired over nearly half-a-century of ICU care have brought us closer to meeting them, as technological advances in instrumentation now effectively put this hard-won knowledge into action. Rising demand in the face of economic constraints is likely to drive future innovations focused on reducing the need for user input, automating multi-element protocols, and carefully monitoring the patient for progress and complications.

## Introduction

Mechanical ventilation is instrumental in the rescue and maintenance of the patient with failing cardiorespiratory function. With passing time, the goals of ventilatory support have been refined to include not only effective life-support, but also minimized iatrogenesis and improved coordination between patient needs or demand and machine-delivered breathing cycles. The capacity of mechanical ventilators to ventilate and oxygenate effectively has steadily improved, while the caregiver has become aware of its potential to cause infection, hemodynamic consequences, and ventilator-induced lung injury. Once an inherently uncomfortable process that invariably required deep sedation and even paralysis to maintain, modern machines provide diverse options to reduce breathing work load, improve comfort, and enhance coordination. In this discussion I recount the important lessons we have learned during the positive pressure ventilation era, describe current developments, and suggest remaining problems and innovative approaches that point toward future progress.

## Mechanical ventilation: a brief look back

Although primitive forms of mechanical ventilation were suggested or implemented in the first half of the 20th century, ventilation with positive pressure emerged as an everyday technology only with the birth of the modern ICU in the early 1960s [1]. About that time, ventilatory equipment transitioned from negative-pressure tanks that surrounded the patient to the familiar positive-pressure machines attached only through the airway and facilitate patient access. At first, the ventilator or respirator was envisioned essentially as a push-pull bellows pump with which to move conditioned gas into and out of the lungs. In the first decades of the 1900s, newly developed electric motor-driven pistons allowed enclosures for the patient's thorax and abdomen but prevented caregiver access without interrupting ventilatory support. Drinker-Shaw and Emerson machines were introduced into medical practice in relatively small numbers around 1930, and these came to be known as iron lungs [2]. By the early 1950s, relatively advanced tank-style ventilators were employed success fully during the polio epidemic; however, these negative pressure devices were cumbersome, worked best when the patient was sufficiently conscious to prevent upper airway closure, and could not hope to support a patient with full-blown oxygenation failure. Spurred by this experience and by two war-time conflicts that occurred in rather quick succession, the value of deploying improved life-support technology became evident for both civilian as well as military applications. The roots of positive end-expiratory pressure (PEEP) and noninvasive ventilation also can be traced back to these early years [3].

The 1960s were a pivotal decade in the development of positive pressure ventilation, influenced by advances in physiology and surgery and the need to address the problems of postoperative atelectasis and the traumatic lung injuries of battlefield conflict. Pressure cycled devices that delivered intermittent positive pressure were utilized on the general wards with the intent of helping a variety of patients breathe more deeply, aiding coughing efficiency, forestalling basilar collapse and improving deposition of therapeutic aerosols. Simultaneously, machines that allowed the inflation and deflation phases to be unlinked (separately regulated) and that were expressly designed for sustained life-support of the critically ill were introduced into the newly formed ICUs [4]. Among the more purpose-refined of these early ICU machines was the Puritan-Bennett^® ^MA-1, introduced in 1967. These powerful units, less bulky and more purpose-designed than some contemporary anesthesia-based alternatives, were innovative and durable. But by today's standards they were inflexible, offered only time-cycled, flow-regulated breathing, and provided simply a calibrated exhalation bellows for tidal volume determination and a needle gauge for airway pressure monitoring. Durable circuits were re-usable, airway suctioning was performed only during ventilator disconnections, flow was not displayed, and key ventilation alarms were attached externally.

Looking back, it is interesting to note that these MA-1 machines offered scheduled sighs to be added when delivering breaths of lower amplitude [5]. Primed by the need to prevent atelectasis in healthy lungs during surgery, large tidal volumes of 10 to 20 ml/kg were the entrenched prescription at that time and normal blood gases were targeted, even in patients with catastrophic respiratory failure [6]. The design engineers were also clearly attempting to mimic natural breathing in their offering of sinusoidal and square wave inspiratory flow patterns - those that are associated with the spontaneous selections made by the normal patient during unassisted breathing and by the patient with serious airflow obstruction. Expiratory retard could be applied in the latter cohort in the attempt to avert tidal expiratory airway collapse and to mimic pursed-lip breathing.

The clinician could manipulate only one variable at a time, so that a change of the imposed breathing pattern required sequential rather than simultaneous adjustment of frequency, flow rate, and tidal volume. Pressure-assisted modes of ventilation suitable for the severely ill were not available. In those early days, PEEP - if used at all - was added externally, using valves with high resistance rather than integrated within the ventilator circuit [7]. The most popular mode of ventilation was assist-control with square wave flow, essentially because it was the only form of triggered assistance available for the adult with critical illness.

In the late 1960s, the syndrome of adult respiratory distress (ARDS) and its treatment by PEEP were described [8,9]. Pediatricians had primed adult intensivists by their experience with surfactant deficiency-caused acute lung injury in neonates, but their well-developed and justified concern for the problems of barotrauma and the use of pressure-based modes of ventilation in this population did not translate into adult caregiving until much later. Intubation for the prolonged periods needed to support respiratory failure using tubes sealed to the airway with high pressure gave rise to serious and often permanent laryngeal and tracheal injuries. Attempts to treat the lung gently during ARDS by undertaking extracorporeal gas exchange proved ill-fated, as the patients rescued with extracorporeal membrane oxygenation were very severely affected and late in their disease course. Materials and techniques of the time inflicted unacceptable injury [10].

Treatment of ARDS was one central driver of new approaches to respiratory failure, but clearly not the only one. How to provide partial support, recondition the respiratory muscles, and gauge readiness of the patient to assume the entire ventilatory workload were also pre-occupying concerns of the day [11,12]. As adult clinicians gained more experience in managing such challenging problems, the need to address them efficiently drove the incorporation of better monitoring as well as the radically new modes of assistance such as (synchronised) intermittent mandatory ventilation and PEEP without assisted breathing [13,14]. Over a relatively brief period of time, microprocessor controls and electronic waveform displays of pressure and flow became embedded into the machines themselves, enabling discoveries related to work of breathing, synchrony, and the effects of adjustments in frequency, PEEP, peak flow, and triggering paradigm on effort and dynamic hyperinflation [15,16].

The importance of improved monitoring and mode flexibility became evident throughout the 1970s and 1980s, as laboratory and clinical investigations revealed the full potential for the ventilator to cause both obvious and hidden forms of lethal injury [17,18]. Awareness of the key roles of maximum transalveolar pressure and high tidal volume led to the approach of accepting higher partial pressure of carbon dioxide (permissive hypercapnia) as a necessary consequence of using smaller and safer tidal volumes to support, first, intubated asthmatics [19] and later those with ARDS [20]. High-frequency jet ventilation and high-frequency oscillation were developed and tested as strategies for limiting the lung-damaging potential of maximum tidal pressure while recruiting the unstable lung units of infants with infant respiratory distress syndrome. Although jet ventilators were available early on, adult use of high-frequency oscillation awaited the development of capable machines in the late 1990s [21]. Inhalation of vasodilatory gas mixtures (nitric oxide) that promoted gas exchange through patent lung units first gained popularity in the 1990s [22].

Pressure-regulated modes of ventilation (pressure support, pressure control, and their modern variants) were developed to address with relative safety the varying flow demands of the patient with cardiopulmonary disease. The ability to respond to the patient's changing flow demands, as well as the need to cycle in timely fashion into the exhalation phase, was introduced to machinery developed in the mid-1970s in the form of pressure support (pressure support ventilation) [21]. At first, time-cycled pressure control (pressure control ventilation) was often implemented as inverse-ratio ventilation in the treatment of ARDS [23], an approach that has since faded from favor. In contrast, pressure support, assist-control, and synchronized intermittent mandatory ventilation with either flow-controlled or pressure-controlled breaths have become entrenched as the flexible standard modes of ventilation for more than 30 years.

Observational studies and clinical trials testing the worth of traditional and innovative approaches to lung protection and gas exchange efficiency characterized scientific efforts in mechanical ventilation through the 1990s and into the first decade of the 21st century [24,25]. Current-generation technology has responded admirably to emerging knowledge concerning iatrogenic upper airway damage, lung parenchymal injury, and the consequences of dys-synchrony [26]. Present-day approaches - for example, proportional assist ventilation and neurally adjusted ventilatory assist - are immeasurably more effective than before, but still need to eliminate imperfect integration with the patient's neural demands and underlying physiologic needs. Safety and coordination remain the frontiers for scientific investigation and technological development in this field.

## Lessons learned

### The invasive interface

Among the first harsh lessons of invasive ventilation was that the protracted presence of an endotracheal tube not only increased the resistance through the upper airway, but also provided a pathway for infection and often damaged irreversibly the delicate tissues of the larynx and trachea. Even today, the problem of airway debris is difficult to contend with, as the biofilm that lines the unperfused endotracheal tube combined with interruption of the mucociliary escalator and a disrupted coughing mechanism predisposes to retention of contaminated airway secretions [27]. Accumulation of airway debris causes increased work of breathing, impaires gas exchange, and predisposes to bronchopulmonary infections. Better materials, lower cuff pressures, and improved nursing practices have addressed some of these problems, but clearly not all of them. In-hospital use of noninvasive ventilation was born from the need to address such issues, and with continually improving interfaces now allows for intubation avoidance, improved sleep quality, and safer treatment of patients with diverse cardio pulmonary problems of moderate severity [28].

### Patient-ventilator interactions

Also learned relatively early in the experience with positive-pressure ventilation was the fact that controlling flow rather than pressure could result in high effort and could predispose to breath timing dys-synchrony [29]. Furthermore, insistence on targeting near-normal pH and partial pressure of carbon dioxide in patients with airflow obstruction often produces dynamic hyperinflation and auto-PEEP [15]. This pervasive gas-trapping phenomenon, which is nonhomogeneously distributed, impairs breath triggering, increases work of breathing, and may impedevenous return. In patients with expiratory flow limitation, counter balancing auto-PEEP with added PEEP can improve the sensitivity of breath triggering, improve the homogeneity of ventilation, and reduce dyspnea without further lung distention, hemodynamic compromise, or disadvantage to the muscles of the respiratory system [30,31].

### Ventilator-induced lung injury

High airway pressures and tidal volumes have been shown to damage both healthy and diseased lungs of laboratory animals since the 1970s. Investigations into the causative relationships among mechanical forces, machine settings and cofactors continues to the present day. It is generally understood, however, that the repetitive application of transalveolar pressures and tidal swings of pressure (driving pressure) that substantially exceed those normally encountered during normal tidal breathing will give rise to hemorrhagic edema and inflammation that mimic ARDS [17]. Sustained re-opening of collapsible lung units that are points of stress focusing is, in general, desirable. But debate continues as to the feasibility and relative importance of fully recruiting all collapsed units, as the latter requires that alveolar pressures do not fall below a high threshold that initiates closure of refractory-dependent units [32]. Because recruiting unstable alveoli can dramatically reduce the incidence of ventilator-induced lung injury, a persuasive rationale exists for recruiting maneuvers, prone positioning, and the early use of high-level PEEP - the latter obligating use of relatively small driving pressures and accepting resultant hypercapnia when necessary.

We have learned only slowly to account for the important influence of the chest wall on measured values of pressure at the airway opening. The lung may thus be exposed to lower or higher transalveolar pressures than suggested by the unmodified plateau pressure or PEEP. Even when considering alveoli in different sectors, stresses and strains upon tissues almost undoubtedly vary greatly, in part because of variations in the environment surrounding those lung regions.

### Complexity and clinical trials

**Figure 1:**
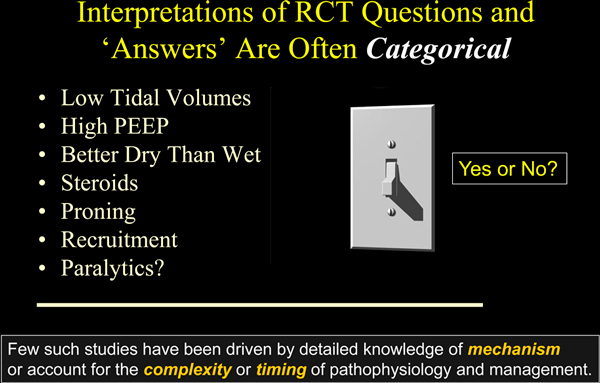
**Dichotomous nature of clinical trials**. The dose, duration, and/or timing of the tested interventions influence their efficacy, so the results and conclusions often should be considered specific to the protocol and tested population, rather than a categorical endorsement or rejection of the tested therapy. PEEP, positive end-expiratory pressure; RCT, randomized controlled trials.

**Figure 2:**
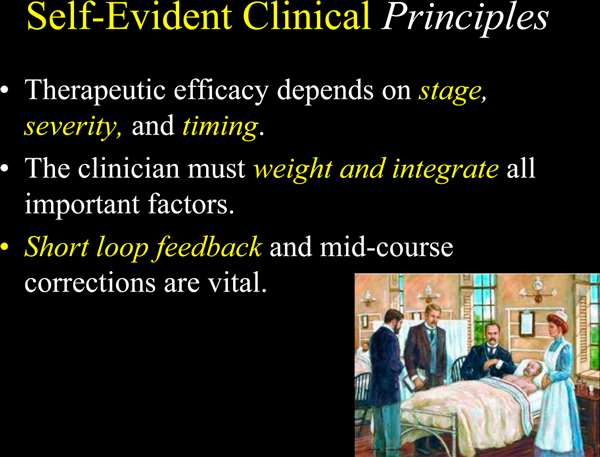
**Useful principles of problem management in critical care practice**.

Few rules governing mechanical ventilation apply across all phases and severities of acute illness; choices must be conditioned by stage and by patient response. Many of the tested questions have sought 'yes or no, toggle switch' answers (Figure 1). Yet even those interventions that seem amenable to such dichotomous testing are nuanced by considerations of their dose, duration, timing of use, and patient selection. Complexity of co-morbidities, timing stages, and co-interventions requires the clinician to weigh and integrate all important factors before making a decision, and then to employ short-loop feedback with frequent mid-course corrections [33] (Figure 2). Knowing these key principles of effective clinical practice, it is wise to remember that few clinical trials have been undertaken with detailed or proven knowledge of the underlying mechanism driving the outcome variable or have accounted for the complexity and timing of pathophysiology and management. As a simple example, none of the multicenter cooperative trials of mechanical ventilation yet conducted has assured passivity of the study cohort, despite the implications of muscular effort for the transalveolar pressures that lie at the root of ventilator-induced lung injury.

Without question, we have learned substantially from the conduct of clinical trials. But, as with physiologic principles gathered from laboratory models of disease, results from population-based clinical trials are only a starting point to guide thinking in many matters related to mechanical ventilation of the critically ill. In many instances, I believe we are well served by unproven experience-based rules (Table 1). Examples abound; high levels of PEEP are relatively helpful in the early stage of ARDS management when the lung is relatively wet and recruitable and benefit outweighs hazard [34,35]. During this initial period of support, recruiting maneuvers (in themselves may be only transiently effective) are often required to set optimal PEEP, which is best selected using deflation characteristics and functional gas exchange responses. Later in the patient's course (or when the lung is poorly recruitable for other reasons), PEEP simply adds to the peak and average airway pressures, both accentuating stresses and strains associated with a given tidal volume and creating deadspace. Advisability of prone positioning may also be time and severity dependent. Meta-analysis of clinical trials data indicates that prone positioning seems to reduce mortality only in those patients who are both severely affected and in the early stage of illness [36]. Persuasive evidence suggests that we are learning similar lessons regarding timing and empiricism when using glucocorticoids [37,38] and recruiting maneuvers [39] in the management of such patients.

**Table 1:** Unproven principles of management for mechanical ventilation

Unproven experience-based rules regarding ventilation support
• Modify therapy according to patient size, physiological demand, and tolerance
• Reduce ventilation intensity as well as demand
• It is transpulmonary pressure that counts
• It is functional response that is important
• Position is an essential tool ... and a hazard
• Recruiting maneuvers often required to set best positive end-expiratory pressure

### Conditional benefits of spontaneous efforts

Another important lesson learned is that there is a need to strike a balance between the benefits of spontaneous breathing and the dangers of oversedation and neuromuscular paralysis. Ventilator-induced diaphragmatic dysfunction should clearly be of concern when fully controlled ventilation is imposed for extended periods [40,41]. Furthermore, unlabored spontaneous patterns of breathing (not accompanied by dyspnea or expiratory muscular effort), appear to be more mechanically efficient than are those administered to a passive patient [42,43]. Yet taking control of ventilation during the earliest phase of life-threatening sepsis and ARDS may enable reductions in potentially damaging mechanical forces arising from high cardiac output and minute ventilation [44,45]. Brief use of paralytics during the most vulnerable early period of illness is not necessarily associated with delayed neuromuscular recovery or ventilator-associated diaphragmatic dysfunction. That being said, it is now strongly suspected that sustained suppression of awareness by large uninterrupted doses of sedatives without periodically returning the patient to consciousness extends the likelihood of prolonged mechanical ventilation, delirium, inability to wean, and consequent adverse clinical outcomes [46].

### Unproven rules of ventilator management

**Figure 3:**
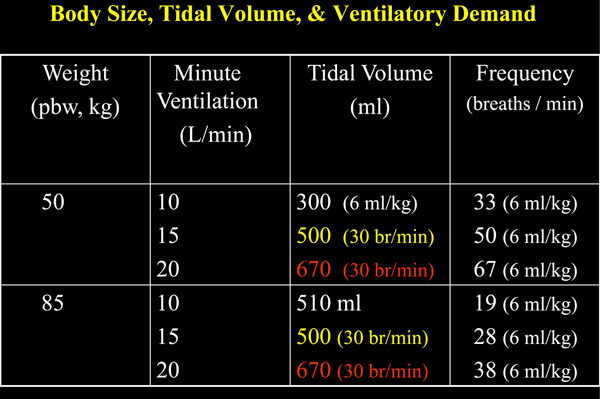
**Influence of minute ventilation on breathing frequency for two patients of differing body size**. Minute ventilation influences on breathing frequency for two patients of differing body size (50 kg and 85 kg). Using the 6 ml/kg predicted body weight guideline, a small patient would be obligated to breathe at an unacceptably high frequency as minute ventilation increases (15 and 20 l/minute). Observing the commonly used upper limit of 30 breaths/minute, tidal volumes far in excess of the 6 ml/kg criterion would be required to ventilate the smaller patient in this higher range (500 and 670 ml, as opposed to 300 ml). br, breaths; pbw, predicted body weight.

Self-evident rules regarding mechanical ventilation have emerged from decades of our collective experience at the bedside. But as yet these rules remain unproven by rigorous clinical trials - and some may never be proven. Ventilatory management of the acute phase of ARDS provides several good examples of our unproven folk wisdom. A major step forward in the prevention of lung damage was to relate tidal volume to predicted (lean) as opposed to measured body weight [47]. Using predicted weight helps scale tidal volume to the underlying anatomical dimension of the lung. Yet the simple rule of 6 ml/kg predicted body weight that has gained traction for protocols used in daily practice is not sufficient for all situations relating to body stature and ventilation demand. The guideline may need to be adjusted upward when patients are small and ventilation demands are high (Figure 3). On the flip side, 6 ml/kg is not always a safe device. Because tidal volume enters only the aerated compartment, it may (depending on compartmental capacity) generate an inadvisably high specific tidal volume and consequently excessive transalveolar pressures and strain during passive inflation. Any inspiratory muscle activity adds further to actual mechanical stress on delicate tissue.

We should also modify therapy according to the patient's physiological need. For example, employing a guideline-approved small tidal volume without reducing a high minute ventilation demand may incur dyspnea as well as inappropriate high breathing frequencies. Whenever possible, we should attempt to reduce the ventilation intensity as well as the patient's demand for support. Reducing agitation, pain, body temperature, and metabolic acidosis are often addressable. Sedation may also be required to tolerate permissive hypercapnia. Refocusing on the pressure difference across the lung is important, as the peak and driving transpulmonary (transalveolar) pressures are those that count with respect to the causation of iatrogenic lung damage [48]. In theory, knowing the functional residual capacity and the transalveolar (as opposed to plateau) static pressure would be necessary to interpret the safety of our tidal volume selection.

Thoughtful clinicians seek ways other than modifying the tidal volume and PEEP to ventilate protectively. From the viewpoint of clinical trial evidence, most methods remain unproven. One aspect of management that may have received insufficient attention in ARDS management is the need to reduce the effects of high flow on tidal shearing forces. Because the baby lung has a reduced number of open airways, flows that would be tolerable in a larger, high-capacity, fully open lung can theoretically result in unacceptable rates of tissue opening. For example, ventilation of 10 l/minute equates to 40 l/minute and an inspiratory average flow velocity of at least double that value in the typical patient whose actual functional residual capacity is reduced to one-quarter of normal. Whereas the open conducting channels may not be directly injured, units that open quickly during inflation may be more vulnerable to epithelial shearing. Moreover, the popularity of pressure control ventilation promotes very high peak inspiratory flows that occur just at the time during which unstable units have yet to be opened. Some experimental evidence in small and large animals strongly implicates high peak flow and delivery profiles as key to generating or avoiding ventilator-induced lung injury [49-51].

**Figure 4:**
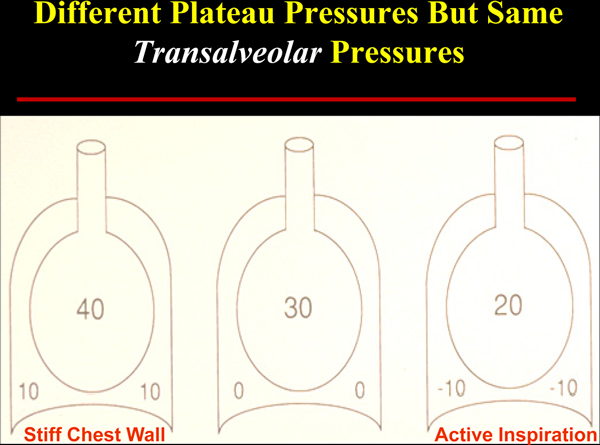
**Effect of chest wall stiffness and active inspiration on plateau pressure**. Although transalveolar pressure and lung dimension are unchanging, airway plateau pressure is strongly influenced by chest wall compliance (left panel) and by inspiratory effort (right panel). Numbers refer to pressures (cmH_2_O) in the respective alveolar and pleural compartments.

Although stretching, shearing, and small airway trauma have been demonstrated to occur when transpulmonary pressures are excessive, tissue tension cannot be directly measured. Unfortunately our reliance on airway pressures alone (PEEP and plateau pressures) - which merge information from all air-containing sectors, are distorted by chest wall stiffness, and are influenced by the presence or absence of spontaneous breathing efforts - glosses over such realities (Figure 4). Experienced clinicians are aware that airway pressures alone may be misleading when the chest wall is stiffened by obesity, surgery, trauma, or disease as well as when the patient makes forceful inspiratory and expiratory efforts. Even measuring transpulmonary pressure with the aid of an esophageal balloon catheter may not be enough [52-54]. A challenging aspect of managing the stresses and strains developed within a mechanically heterogeneous lung is the amplification (or stress focusing) that occurs at the interfaces between fully open and closed lung units [55].

### Well-intentioned but dysfunctional practices

It is humbling to consider that practices which have gone many years unquestioned might contribute to the generation or extension of the primary disease we are trying to resolve. Acute illness progresses through phases. In general, we have not taken into account that the underlying pathophysiology varies with disease stage, and that such physiological differences should factor strongly into our management. Here is one possible example: in the early stage of pneumonia treatment, the intubated patient is typically hydrated, given antibiotics, and repositioned frequently to avoid decubitus ulceration of the skin and to improve comfort. We often encourage such patients to breathe spontaneously, with each forceful call for and assisted breath resulting in the delivery of relatively high transpulmonary pressure and tidal volume. PEEP is not considered helpful in lobar disease unless maintenance of adequate oxygenation requires it. With the patient's ability to expel secretions impaired by intubation, we suction the airway frequently and promote coughing in the process. Yet we may need to rethink our approach in this earliest stage of pneumonia [56].

**Figure 5:**
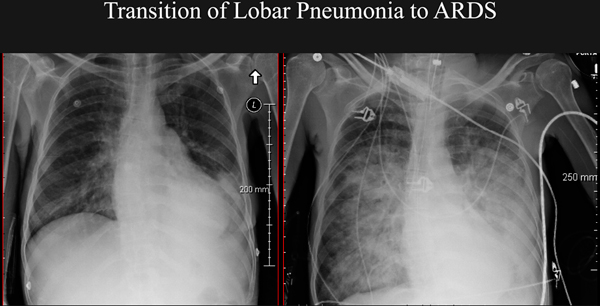
**Transition to adult respiratory distress syndrome from left lower lobe pneumonia following airway intubation**. Transition to adult respiratory distress syndrome (ARDS; right panel) from left lower lobe pneumonia (left panel) over the 18 hours following airway intubation and conventional management in a 28-year-old woman without heart disease.

**Figure 6:**
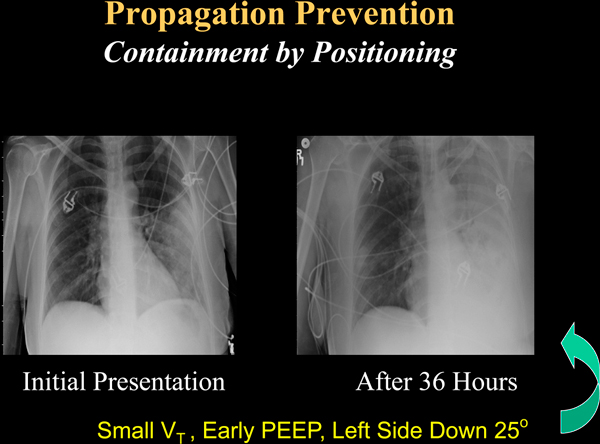
**Progression of left lower lobe pneumonia treated consistent with containment of mobile airway biofluids**. Sequence of progression in a 31-year-old woman with left lower lobe pneumonia treated by principles consistent with containment of mobile airway biofluids. Although infiltrates spread through the dependent left side over a 36-hour period, adult respiratory distress syndrome did not develop. PEEP, positive end-expiratory pressure; V_T_, tidal volume.

Thin proteinaceous and mediator-laden fluids with great potential for spreading through the airway network characterize this earliest phase [57,58]. During these early post-intubation hours these mobile biofluids can extend injury, cause metastatic lobar infection [59], and even propagate a process that culminates in diffuse lung injury that we label primary ARDS (Figure 5). Before propagation happens and focal lung disease becomes generalized, implementing moderate PEEP to peripheralize liquid, small tidal volumes, inhibited coughing, enforced quiet breathing, and dependent positioning of the affected side may be the most rational strategy to contain the pneumonia to its region of origin [55] (Figure 6). Later, the well-intentioned suctioning, movement, and lower PEEP with higher tidal volume are perfectly rational in helping to expel the thickened and less dangerous biofluids known as sputum. One must emphasize that this 'propagation prevention with injury avoidance' hypothesis is mechanistically plausible but unproven. A clinical trial to determine its validity would be informative.

## Recent progress and future prospects

### Emerging technologies

Important challenges remain in current practice (Table 2). Although we have learned important lessons much too slowly regarding the dangers of protracted endotracheal intubation, ventilator-induced lung injury, sedation issues, and breathing dys-synchrony, our cumulative experience has given rise to advances with potential for better care of the ventilated patient. Greatly improved noninvasive ventilation may obviate the need for more invasive approaches in many patients. For the foreseeable future, however, intubation will continue to be required to protect the airway, to extract retained secretions, to allow deep sedation, and to control ventilation for purposes of manipulating the airway or performing cardiothoracic surgery.

**Table 2:** Continuing challenges relative to ventilator management

Address and minimize heterogeneity of mechanics
Minimize demands for ventilation
Optimize sedation and comfort
Minimize time on ventilator
Coordinate appropriately with ventilatory drive
Automate adjustment for changing patient needs
Safely help the patient adapt to the disease
Prevent infection and adult respiratory distress syndrome

Secretion retention will therefore probably remain a vexing source of complications so long as invasive intubation is required. The unperfused biofilm that lines the tube is inaccessible to host defenses, providing a safe haven for large infective inoculums to form and later seed the lung. Nonetheless, approaches that minimize or remove the infective endotracheal biofilm, visualize the proximal airways, reduce secretion impaction, and assist with sputum elimination by attention to inspiratory flow modification, percussive vibration of the air column, and mechanically aided coughing promise to minimize secretion-related complications [60-64].

Genuine progress has also been made in the attempt to link appropriate patient demands for ventilatory assistance with synchronous triggering and power. Initial benefits from pressure support and pressure control have paved the way for recently released innovations such as proportional assist ventilation and neurally adjusted ventilatory assist [65,66]. With better monitoring of mechanics and gas exchange, automated goal-directed algorithms integrated into the machine circuitry may enable automated upregulation and downregulation of power assistance, fraction of inspired oxygen, and PEEP, according to demands and patient response. These algorithms have only recently gained traction in the clinical setting but clearly are steps in the right direction.

**Figure 7:**
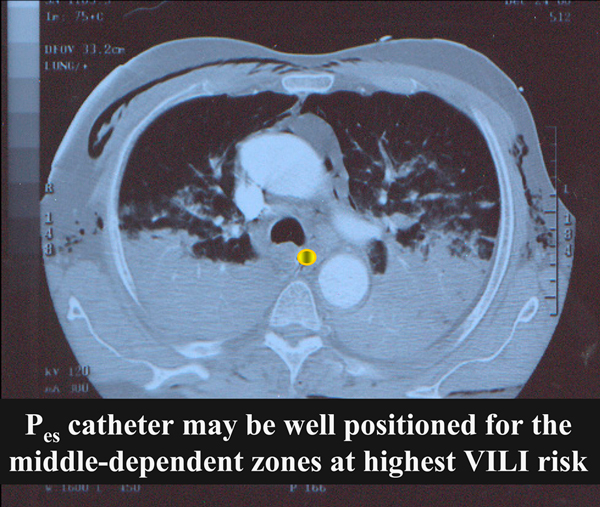
**Position of esophagus between open and closed lung units in adult respiratory distress syndrome**. Position of esophagus in relation to the interface between open and closed lung units in a patient with early-stage adult respiratory distress syndrome. Regional pressure recorded within the esophagus (P_es_) and along the sagittal and coronal planes that intersect it may be representative of pressures relevant to the stress-focused and relatively unstable units at the aerated and airless interface. VILI, ventilator-induced lung injury.

Concerns regarding ventilator-induced lung injury continue, of course, but deployment of the laboratory-proven and venerated esophageal balloon monitoring of pleural pressure may now enable routine determination of transpulmonary pressure - a value that comes a step closer to the calculation of effective stress upon the lung itself during spontaneous breathing and that helps select the PEEP that must be applied to keep it positive so as to avoid collapse [54] (Figure 7). Direct measurement of functional residual capacity allows estimation of the size of the baby lung, which does not always coincide with estimates based on transpulmonary pressure [67].

Regarding the force amplification at points of stress focusing, there is still a considerable gap that needs closure. Here too, however, tools needed for regional and dynamic monitoring of the heterogeneous lung are becoming available in the form of bedside regional imaging methodologies such as electrical impedance tomography and ultrasonic probing of the diseased lung [68,69]. These methods currently offer impressive qualitative insights, even if they lack quantitative precision at this time.

Reducing the need to ventilate and to generate high pressures for ventilation, lung recruitment, and oxygenation with the patient remaining fully conscious and with spontaneously breathing has been a clear but elusive goal that is now much closer to widespread implementation. Prudently administered pharmaceuticals and judicious use of renal replacement therapies applied in a timely fashion can dramatically lower ventilatory demand and improve the efficiency of oxygenation. Moreover, a variety of bedside adjuncts, both extracorporeal and intravascular, assist in eliminating carbon dioxide and replenishing the oxygen content of venous blood returning to the heart [70,71]. Such methodologies were urgently and successfully applied in the treatment of severely affected patients with H1N1 lung injury [72].

### A few predictions

As we progress through this early part of the 21st century, emerging economic realities will help drive our approach to bedside care (Table 3). We will probably have fewer personnel deployed per patient for both observation and intervention. Caregivers will be aided by electronic information handling, but it is unclear at this time how well prepared the individual caregiver will be to think analytically when managing the required information stream and knowledge base. Hospital administrations are likely to demand faster hospital throughput while emphasizing the priorities of safety, timely intervention, and avoidance of complications. Aggressive attempts will be made to protocolize many aspects of care. Such needs may spawn a variety of future innovations in mechanical ventilation (Table 4). Smarter machines will reduce the need for user input and monitoring. Flexible equipment will be needed to address patients of all sizes and conditions and to apply multi-element protocols automatically while carefully monitoring the patient for unanticipated deviations and complications. To make such automation safely possible, advanced ventilators will not only monitor pressures and flows, but also exhaled gas analysis and inputs from the hemodynamic side. I anticipate that machines of the future will be goal-directed and self-adapting, fully capable of integrating mechanics, gas exchange, and cardiovascular information to achieve the clinical targets. Remote reporting and machine adjustment are a clear and natural evolution. Past lessons and future needs will shift the ventilatory paradigm (Table 5).

**Table 3:** Emerging economic realities related to critical care that must be confronted in the future

• Fewer personnel per patient
- Observation
- Intervention
• Faster hospital throughput
• Increased needs for:
- Safety
- Timely intervention
- Quicker assessment of therapy
- Decision support

**Table 4:** Future innovations in mechanical ventilation

Goal-directed self-adaptation
Reduction of ventilatory demand
Remote reporting and machine adjustments
Patient-guided control
Improved secretion management
Bedside lung imaging
Multisystem integrated monitoring

**Table 5:** The shifting paradigm relating care delivered to the mechanically ventilated patient

Observe time sensitivity of treatments
• Paralytics
• Prone positioning
Give ventilation control to patient (?)
Reduce demands
Revise targets
• Monitor the key variables
Adapt to abnormal physiology
Exchange gas without mechanical ventilation

## Conclusion

Unchanging needs for providing effective life-support with minimized risk and optimized comfort have been, are now, and will remain the principal objectives of mechanical ventilation. Important lessons acquired during almost half a century of ICU care have brought us closer to meeting these elusive goals. Perhaps the over-arching theme of our education, however, is that a solid understanding of organ system physiology is the fundamental and irreplaceable tool for guiding our progress.

## Abbreviations

ARDS: adult respiratory distress syndrome; PEEP: positive end-expiratory pressure.

## Competing interests

The author declares that they have no competing interests.

## Declarations

This article has been published as part of *Critical Care *Volume 17 Suppl 1, 2013: Future of Critical Care Medicine. The supplement was proposed by Fresenius Kabi based on presentations from the 'Future of critical care medicine (FCCM) 2012: Today's practice and a look to the future' symposium. Articles were commissioned by the journal, were independently prepared by the authors and have been peer reviewed by the journal. The supplement was edited by John Marini, who declares that he has no competing interests. Publication of the supplement was supported by Fresenius Kabi.
